# Robust Cluster Prediction Across Data Types Validates Association of Sex and Therapy Response in GBM

**DOI:** 10.3390/cancers17030445

**Published:** 2025-01-28

**Authors:** David L. Gibbs, Gino Cioffi, Boris Aguilar, Kristin A. Waite, Edward Pan, Jacob Mandel, Yoshie Umemura, Jingqin Luo, Joshua B. Rubin, David Pot, Jill Barnholtz-Sloan

**Affiliations:** 1 Thorsson-Shmulevich Lab, Institute of Systems Biology, Seattle, WA 98109, USA; 2 Trans Divisional Research Program, Division of Cancer Epidemiology and Genetics, National Cancer Institute, Bethesda, MD 20892, USAjill.barnholtz-sloan@nih.gov (J.B.-S.); 3 Global Oncology Research & Development, Daiichi-Sankyo, Inc., Basking Ridge, NJ 07920, USA; 4 Department of Neurology and Neurosurgery, Baylor College of Medicine, Houston, TX 77030, USA; 5 IVY Brain Tumor Center, Barrow Neurological Institute, Phoenix, AZ 85013, USA; 6 Department of Surgery, Division of Public Health Sciences, Washington University School of Medicine, St. Louis, MO 63110, USA; 7 Siteman Cancer Center Biostatistics and Qualitative Research Shared Resource, Washington University School of Medicine, St. Louis, MO 63110, USA; 8 Department of Pediatrics, Washington University School of Medicine, St. Louis, MO 63110, USA; 9 Department of Neuroscience, Washington University School of Medicine, St. Louis, MO 63110, USA; 10 General Dynamics Information Technology, Falls Church, VA 22042, USA; 11 Center for Biomedical Informatics and Information Technology, National Cancer Institute, Bethesda, MD 20892, USA

**Keywords:** clustering, disease subtyping, machine learning, glioblastoma multiforme, GBM, feature engineering, gene expression signatures, female

## Abstract

Background: Previous studies have described sex-specific patient subtyping in glioblastoma. The cluster labels associated with these “legacy data” were used to train a predictive model capable of recapitulating this clustering in contemporary contexts. Methods: We used robust ensemble machine learning to train a model using gene microarray data to perform multi-platform predictions including RNA-seq and potentially scRNA-seq. Results: The engineered feature set was composed of many previously reported genes that are associated with patient prognosis. Interestingly, these well-known genes formed a predictive signature only for female patients, and the application of the predictive signature to male patients produced unexpected results. Conclusions: This work demonstrates how annotated “legacy data” can be used to build robust predictive models capable of multi-target predictions across multiple platforms.

## Simple Summary

Experimental platforms produce highly different types of data, and with lightweight feature engineering, it is possible to construct robust predictive models to apply across data modalities. In this work, using predefined sample clusters, we developed a feature set that allowed us to train a machine learning model with older gene expression microarray data to make predictions on more recent RNA-seq data. Additionally, since the model is highly interpretable, we could make and test hypotheses on single-cell data. This work strengthened evidence for a female-specific gene expression pattern that is associated with better overall survival outcomes.

## 1. Introduction

In biomedical research, a great deal of effort is put towards disease subtyping, defining patient groups through factors that include disease etiology, developmental trajectory, or some combination of biological attributes such as gene expression signatures [1]. Disease subtypes that can be consistently identified are helpful in selecting efficacious therapies [2].

However, biomedical research continues to transition towards new technologies and more advanced assays that depend on new ways to make fundamental measures [3]. These come with new file formats, new methods of data preprocessing and normalization, and with a general shift in attention from the research community [4]. As a result, the vast library of research products stored in databases like NCBI GEO, which were often produced with older technology —in this case, gene expression microarrays— have been subsumed by newer techniques like bulk RNA-seq and more recently, single-cell RNA-seq, not to mention the ever-growing landscape of spatial sequencing techniques [5].

Tools and techniques that can act as bridges are required for taking the results and data from the past—which is still highly valuable—and bringing it forward into contemporary research [6]. Many times, with some lightweight feature engineering, these studies can still be used to train modern machine learning models that are able to operate across data modalities. In this work, we used a set of gene pair features to identify patient cluster labels, regardless of the data processing pipelines and platforms.

To demonstrate the cross-modality analysis, we focused on glioblastoma multiforme (GBM), an incurable aggressive brain cancer [7]. At the most coarse level, it has been subtyped into two main branches based on the presence of a common somatic mutation in IDH1 (isocitrate dehydrogenase) [8,9].

In 2019, Yang et al. discussed a strong sex-dependent effect in therapeutic response [10]. Using a sample clustering technique, the authors defined five male clusters [mc] (termed “mc1” to “mc5”) and five female clusters [fc] (termed “fc1” to “fc5”) separately and identified one male cluster (“mc5”) and one female cluster (“fc3”) that clearly had a better response to the standard treatment. Interestingly, the two groups showing better survival characteristics were characterized by sex-dependent non-overlapping gene signatures. The patient clustering was validated through the use of additional array-based gene expression datasets.

In this work, we trained ensemble-based machine learning models on TCGA gene microarray data [11] annotated with cluster labels from Yang et al [10]. Validation was performed on three additional microarray datasets, also shown in the original Yang et al. [10] study, which includes REMBRANDT [12,13,14]. Additionally, the model was applied to RNA-seq data using matched TCGA data [15], data from the University of Michigan, the University of Texas Southwestern, and Baylor College of Medicine; the model was sequenced by Tempus (referred as the TEMPUS data in this manuscript) and data from CPTAC3 [16]. Furthermore, we tested hypotheses on single-cell RNA-seq data, uncovering the cell type populations that are potentially involved in the predictive gene expression patterns [17].

For the female cluster, fc3, with only 10 gene pairs per cluster, we are able to make satisfactory multi-modal predictions on patient cluster labels which potentially predict a favorable response to therapy. We found that the female signature was able to be replicated in a recent study using RNA-seq data from the TEMPUS GBM patient cohort and found the predictive pattern only in tumor cells using scRNA-seq.

## 2. Approach

### 2.1. Statistical Decision-Making Approach

The goal was to construct a predictive model that was robust to noise and applicable to various experimental platforms, specifically microarray platforms and RNA-seq. To do that, we utilized a feature pair approach which has been shown to be highly useful in constructing robust predictive models [18,19,20]. The approach is fundamentally based on making predictions by comparing two values, in this case, two gene expression values. For example, if we had a gene pair predictor made up from two genes, gene A and gene B, we could formulate a model as a conditional statement, if gene A > gene B, then patient group X, else patient group Y.

This statement expresses that for a given patient, if the expression of gene A is greater than gene B, then we classify the patient into group X. This implies that the gene expression ordering is reversed in patient group Y (gene B > gene A). In use, when making the comparison, the data becomes “within sample normalized” and makes it comparable across patients without batch effects or requiring preprocessing.

Over time, this approach has been expanded to include the use of multiple feature pairs, multi-class targets, and has even been integrated with other machine learning techniques such as SVMs, decision trees, and random forests [21,22,23,24,25,26].

In this study, we used the R package “robencla” (ROBust ENsemble of CLAssifiers, release version 0.3.4), which makes multi-class predictions with an ensemble of XGBoost classifiers [27]. The model was initially developed in the context of predicting immune subtypes as part of the Immune Landscape of Cancer [28]. The software takes a list of gene pairs for each cluster and builds an ensemble of binary classifiers for each target (cluster). Ensembles produce a score for each potential cluster assignment and then feed the scores to a final predictor to make the “Best Call” for each sample.

### 2.2. Feature Selection for Cluster Prediction

In the Supplemental Materials from Yang et al. [10], cluster assignments were provided as lists of TCGA sample barcodes. Additionally, from the supplement, associated genes for each cluster (fc1–fc5 and mc1–mc5) were acquired as gene symbols.

Initially the gene lists from Yang et al. [10] were used to make cluster label predictions. However, it was found that some genes were included in multiple cluster signatures, and some clusters have far greater numbers of genes, making for an off-balance feature set (Supplementary Figure S1) and ultimately moderate prediction performance. For example, in the least difficult classification problem, cross-validation on TCGA samples simply using the genes as features, it was observed that males had an average F1 score of 0.7 (F1 is the harmonic mean of precision and recall with a range of zero to one corresponding to poorest to best performance). Female patients had an average F1 score of 0.75. These results indicated that further refinement was needed.

In order to work towards a more optimal set of features, we derived a scoring function where for each patient cluster, a ranking of gene pairs was produced. The scoring function is an aggregate of a few parts. First, common to paired feature prediction schemes, the most important metric is usually a cluster purity metric considering the difference in proportions, i.e., the difference in the percentage of samples with the gene pair correctly ranked between two given sample groups.

(1) $$\mathrm{Proportion}\;\mathrm{Difference}\;(\mathrm{PD})=P_{s\;\in \;S}\left(G_{i}<G_{j}\;\right)-P_{t\;\in S\;}\left(G_{i}<G_{j}\;\right)\;$$

where *S* is the total set of samples, *s* are samples in a given cluster *C*, *t* are samples not in cluster *C*, and *G_i_* and *G_j_* are gene expression values. At the extremes, the two genes are consistently in one configuration; one gene is always more highly expressed than the other within the cluster, and the pattern is reversed in all other clusters.

However, another consideration beyond the cluster purity of the gene pair relation should be the effect size, namely the expression space distance between the two genes. The greater the distance between the average expression levels, the more likely the relationship would hold as we consider more and varied types of data from alternate platforms. With that in mind, we define the score function to take into account the proportion difference as well as the magnitude of difference between genes inside the cluster and outside the cluster.

*Gene Pair Score* = |*PD*| ∗ (−1 ∗ *Q* ∗ *R*) (2)

where *PD* is the absolute value proportion difference (Equation (1)), *Q* is the average difference between the gene pair within patient group *X*, and *R* is the average difference between the gene pair not in patient group *X*. To produce a high score, *Q* and *R* will have different signs, indicating the expression pattern is reversed.

**Figure 1:**
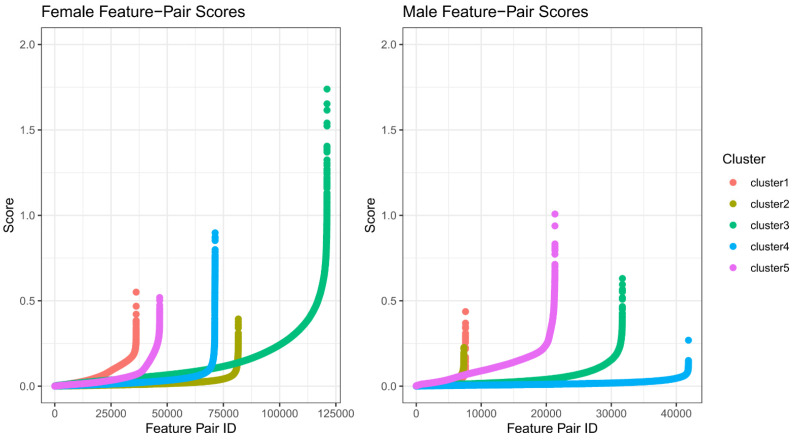
The gene pair scoring metric ranks gene pairs. Each gene pair receives a score indicating whether it is expected to be useful as a predictive feature. After sorting, each point indicates a gene pair score per cluster label using TCGA-GBM array data (each showing one cluster vs. all others). Sorted scores from female patient data (left) show a very strong signal from the protective cluster, fc3 (green), while male patient data provide smaller scores overall but with the protective cluster, mc5, showing the highest feature pair scores (purple).

After ranking the gene pairs for each patient group (over all possible gene pairs), a count of gene pairs with a *gene pair score* greater than 0.5 demonstrated large differences between the “protective cluster” and the others. Indeed, the signal is quite strong in this group, especially in the female patient group, fc3 (Figure 1). This also shows that while identifying members of the protective cluster will be tractable, identifying members outside this group will be more challenging.

Later, after making predictions on the validation array data (Section 3.3), the feature score metric was updated by including the proportion difference (*PD*, Equation (1)) from the predicted cluster labels of the validation array data (*PDval*). This ensured that selected features would be concordant between the two data sources. Finally, some manual curation was performed to consider the correlation between array data and RNA-seq for selected genes.

*Updated Gene Pair Score* = |*PD_TCGA_*| ∗ |*PD_val_*| ∗ (−1 ∗ *Q* ∗ *R*) (3)

### 2.3. Robust Ensemble Model Training

The robencla R package (v0.3.4) was used in training and making predictions. The package provides convenience functions for predicting multi-label targets using ensembles of XGBoost classifiers. Each cluster is predicted with a specific stack of binary classifiers, where each member of the ensemble was trained with a subset of the data. The median of ensemble predictions is taken, and the resulting set of predictions are fed into a final XGBoost ensemble to call the label. A grid search using cross-validation on TCGA was used to determine model parameters. The full parameter set used is found in Supplementary Table S1.

## 3. Results

### 3.1. Model Cross-Validation Using TCGA-GBM Array-Based Data

**Figure 2:**
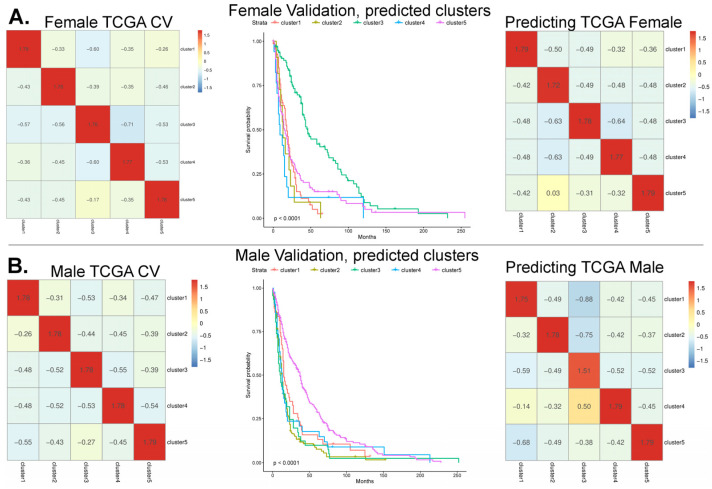
Cluster label predictions on gene microarray data. (**A**, **Left upper panel**) Column-scaled heatmaps show the results from TCGA-GBM array cross-validation. True labels are in columns (c1-c5, **left** to **right**) and predicted cluster labels are in rows. (**A**, **Upper center panel**) Survival curves stratified with predicted cluster labels on the female patient validation microarray data; the results show fc3 as protective (*p* < 0.0001). (**A**, **Upper right**) A model trained on the validation array data with predicted cluster labels shows high-quality predictions back to known TCGA-GBM cluster labels. (**B**, **Lower center** and **right**) Survival curves show mc3 predictions as protective and models trained on validation array data were able to recapitulate known TCGA-GBM labels (*p* < 0.0001). Here, male results were generated with the refined feature set (Section 3.3).

The task of building a robust classifier in this context is challenging due to the small number of examples per cluster. In TCGA-GBM, there are a total of 220 male samples, with 36 in cluster mc5, and for female patients, there are a total of 140 samples, with only 14 in the protective cluster fc3. However, we observed a strong signal in the female dataset, which led to better predictive performance compared to the male dataset. Using a set of 10 gene pairs per cluster, we were able to accurately predict the cluster label of TCGA-GBM patients with a 10-fold cross-validation (Figure 2A). Classification metrics are found in Supplementary Table S2.

For TCGA-GBM males, the average cross-validation accuracy and F1 average across the five clusters were 0.85 and 0.840, and the sensitivity and specificity for mc5 was 0.86 and 0.96, respectively. For females, the average accuracy and F1 was 0.82 and 0.82, and the sensitivity and specificity for fc3 was only 0.79 and 1.0. For fc3, the sensitivity was relatively low, but we must consider the extremely small sample size (14 samples in fc3) and the excellent specificity.

During the work, we found that parameter changes in the underlying XGBoost classifiers led to negligible changes in performance, which signaled the robust nature of the classifier. Within the model, there was also a steep drop in information gain beyond the first five gene pair features.

In the initial feature set, for males, the most informative feature was the gene pair CENPF-NRGN (0.29 log2bits information gain), followed by SERPINI1-RRM2 (0.18 log2bits information gain), and for females, the most informative feature was RBP1-BMP2 (0.43 log2bits information gain), followed by SCN3A-DYNLT3 (0.34 log2bits information gain).

### 3.2. Validation of the Array-Based Model with Three Additional Datasets

In Yang et al.’s [10] work, additional datasets were used to validate protective clusters. Following that, we also added three additional gene expression microarray datasets from GSE13041, GSE16011, and REMBRANDT (GSE108474). This produced an array validation set of 536 male patient samples and 304 female patient samples.

To judge the classifier performance on the validation array data, two approaches were taken. First, we consider if the protective clusters have clearly separated and have better survival curves compared to the other clusters, and secondly, we consider if the quality of the cluster labeling is enough to recapitulate the Yang et al. [10] clusters in TCGA-GBM from a model trained on the validation array data with predicted clusters.

In the case of female patients, using the top 10 ranked gene pairs, the predicted fc3 cluster showed strongly separated survival curves (*p* < 0.0001) compared to all other clusters; the resulting survival plot appeared similar to that of Yang et al. [10]. In the REMBRANDT study, the patient diagnosis in predicted cluster fc3 showed that most patients were previously diagnosed with astrocytoma (40%) and oligodendroglioma (34%) compared to GBM (7%), mixed (6%), or other (13%). The validation array data with predicted cluster labels were then used to train a new model, making predictions back onto TCGA data with an overall accuracy of 92.8% [10], demonstrating that the most important patterns had been sufficiently captured.

### 3.3. Feature Refinement Improves Generalizability of Classification

For male patients, the classification performance on the validation array data appeared quite poor. No samples were predicted for mc2 and both clusters mc3 and mc5 were separated from mc1 and mc4, with better survival.

These results suggested that the initial feature selection ranking could be improved. When the proportion difference (*PD*) of important feature pairs was compared between TCGA data and the validation array data, it was observed that some features were discordant across the datasets. Some feature pairs that held a high importance ranking when predicting clusters in TCGA had no importance on the validation array data.

**Figure 3:**
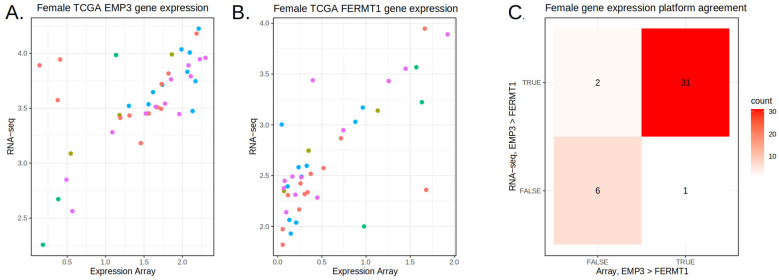
Comparison of gene expression across platforms. The gene pair expression of EMP3-FERMT1 is shown in dimensions including the expression array (x axis) and RNA-seq (y axis) for TCGA-GBM. Colors indicate cluster labels per sample. Although the comparison between array and RNA-seq for EMP3 (**A**) and FERMT1 (**B**) appears highly noisy, in (**C**), the inter-platform gene pair agreement is highly consistent for EMP3 > FERMT1, demonstrating the effectiveness of gene pairs.

To overcome this, we updated the feature scoring function to “up-rank” features with similar proportion differences (*PDs*) between TCGA and the validation array data. This was performed by computing the *PD* using predicted labels and including the validation array data *PDs* into the scoring function. When the expression values for matched TCGA samples were compared between array and RNA-seq, there was some concern about the seemingly high level of technical noise (Figure 3). It has been previously reported that at the low end of the dynamic range in microarrays, there can be a significant amount of noise [29,30,31]. However, when we compared the two platforms using the gene pair comparison (gene A > gene B), we found a consistent relationship. But to again improve the generalizability of the feature set, we further filtered gene pairs by considering the correlation between the array and RNA-seq (Supplementary Figure S2).

For male patient samples, the initial feature set did not perform well in terms of the expected survival curve patterns and required over double the number of features compared to the female patient data. But after the feature refinement, 24 gene pairs were found to produce a validation survival curve that approximated what is shown by Yang et al. [10], with clearly separated protective clusters (*p* < 0.0001). Additionally, the learned pattern was enough to transfer the labels back to the initial TCGA training set with 85% accuracy.

However, the distribution of male patient diagnosis across predicted cluster labels was quite different compared to the female patient group. The REMBRANDT diagnosis counts were now lower in oligodendroglioma and higher in GBM; the proportions were astrocytoma (39%), GBM (36%), oligodendroglioma (10%), mixed (3%), and other (11%).

### 3.4. Prediction on the TEMPUS and CPTAC3 RNA-Seq Datasets

GBM RNA-seq were acquired from the University of Michigan, the University of Texas Southwestern, and the Baylor College of Medicine and processed by Tempus Labs (this collective set of data is referred to as the TEMPUS data in the manuscript). These data are independent of that utilized by Yang et al. [10] Using the refined feature sets, the models were trained on TCGA array data and used to predict cluster labels on TEMPUS RNA-seq data. In this case, the data were made up of 86 female patients and 150 male patients.

**Figure 4:**
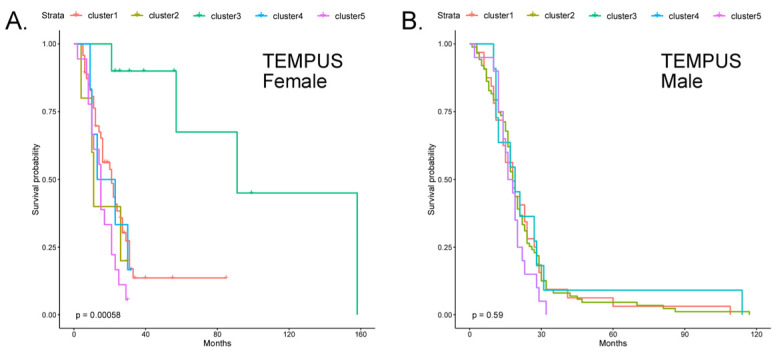
Survival curves on TEMPUS RNA-seq data. Using TCGA-GBM and the refined feature set, models were trained and used to predict cluster labels on (**A**) female and (**B**) male patient data from TEMPUS.

Both the initial feature set, selected only through the scoring metric, and the more refined features made suitable predictions on TEMPUS data (Supplementary Figure S3). When the refined feature set was used to predict cluster labels on the TEMPUS cohort, similar to the validation array data, the fc3 was distinct (log-rank *p*-value = 0.0003) and showed increased survival times compared to the other clusters, which closely grouped together (Figure 4). The distribution of samples to cluster labels was not evenly dealt, with few samples assigned to clusters fc2 and fc4. The male patient samples, on the other hand, did not show any difference in the survival curves across the predicted clusters. When the female predictive features were applied to male TEMPUS data, there was not any difference in the survival curves observed.

Finally, in one last experiment, CPTAC3-GBM RNA-seq data were acquired through the BigQuery tables of ISB-CGC, producing a dataset of 44 female patient samples and 55 male patient samples [32,33]. In this case, the refined features produced quite noisy and poor results.

When the clinical data of the predicted fc3 patients in TEMPUS and CPTAC3 were considered, it was found that all cancers harbored IDH1 mutations, a frequent somatic mutation commonly found in acute myeloid leukemia (20%), cholangiocarcinoma (20%), chondrosarcoma (80%), and low-grade gliomas (80%) [9]. It is likely that since in the CPTAC-3 GBM cohort, there were only a few patients with the IDH1 variant, the results are reasonable. Given that we see better patient survival predicted only in female patients, this appears to indicate the predictive signature is female-specific. If the signature was simply predictive of IDH1 status or survival but was not sex-specific, then we would expect that applying the female model (with female gene signatures) to male IDH1 mutant patients would place IDH1 mutants into fc3, but instead, they are predicted as members of fc4 and fc5, showing the signature as sex-specific.

### 3.5. Aggregating Information Rankings Across Datasets

In the trained robencla model, each predictive feature is ranked with a median amount of information gain. To aggregate the feature rankings across data sources, models were trained using either the given cluster labels (for TCGA-GBM) or predicted cluster labels (for validation and TEMPUS), and the top 10 most informative gene pairs from each model and cluster were collected from the four datasets.

**Figure 5:**
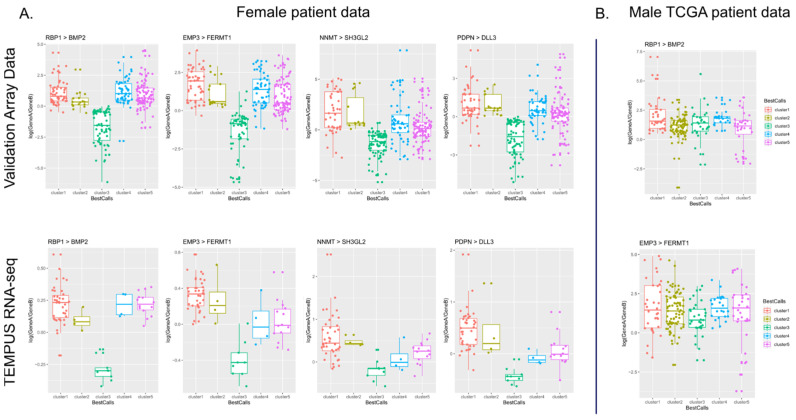
Demonstrating concordance in gene expression ratios across platforms. (**A**) The upper row shows log(gene A/gene B) for each predicted cluster from the validation array data, while the lower row shows log gene ratios from TEMPUS RNA-seq predicted clusters. The pattern of up and down expression ratios is consistent across platforms (array vs. RNA-seq) and cohorts (REMBRANDT vs. TEMPUS), showing the robustness of the selected features. The predictive pattern for the same gene pairs is not observed in male patient data (**B**).

It was observed that often the prediction can be dominated by only a few highly consistent features within a dataset but then carries less predictive power in other datasets. For example, this is evident in the most informative feature found for cluster fc1 in TCGA-GBM, TAP1-FCGBP (median gain 0.161). This feature, while predictive in TCGA-GBM, is not found in the informative lists across any of the other datasets, whereas the third most informative feature for TCGA-GBM, CD46-PDGFRA, is found in the informative lists of all four datasets. From 55 informative features in the TCGA, 11/55 features were only found in TCGA trained models, 15/55 were found in one additional dataset, 13/55 were found in two other datasets, and 14/55 features were found across all datasets. For these highly predictive gene pairs, the expression pattern can be seen in both the validation array data and RNA-seq data (Figure 5).

To aggregate a compact feature set, we selected features that were found in the informative lists of at least three datasets, which typically comprised 27 gene pairs, with about five representing each cluster. The feature set showed good predictive power in both the validation array data and the TEMPUS RNA-seq data (Table 1).

### 3.6. Single-Cell RNA-Seq Provides the Source of the Signal

We hypothesized that if this gene signature is both sex-specific and closely associated with the IDH1 mutation, then it should be visible on the single-cell level by subsetting patients based on these characteristics and checking for the expected expression pattern.

**Figure 6:**
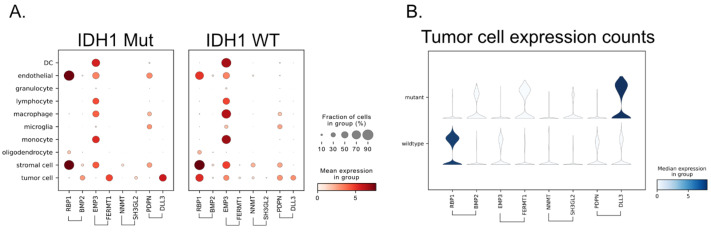
The predictive pattern of gene pair expression is observed in single-cell RNA-seq. (**A**) Each dot shows the percentage of cells expressing a gene, while the color indicates the median expression level. Comparing tumor cell expression patterns between IDH1 mutant to wild type shows the predictive pattern of feature pairs. (**B**) After subsetting to tumor cells only, the predictive expression pattern is again observed.

In Andrieux et al.’s work, the authors have provided harmonized single-cell RNA-seq datasets that are annotated with clinically related features such as IDH1 status [34]. In total, there were 115,673 cells from female glioma patients and 23,552 cells from female patients that were of the IDH1 mutant, the remainder being of the IDH1 wild type. By comparing the summarized expression between IDH1 mutants and wild types per cell type, we can start to get a sense of potential sources of the gene ranking patterns (Figure 6). In this case, when we consider gene pairs such as EMP3-FERMT1 and RBP1-BMP2, we see that while EMP3 is expressed across many immune cells and RBP1 is expressed in endothelial cells, stromal cells, and oligodendrocytes, there is only one major cell type where we see the binary predictive pattern, namely tumor cells. This essentially rules out other sources of the predictive pattern, such as those possibilities in the tumor microenvironment.

## 4. Discussion

The NCBI GEO data repository has continued to grow over its 24-year life [5]. In 2023, it was reported that the rate of submitted data is doubling every 5 years, and it contains over 200,000 studies. Over that amount of time, the research community has experienced a true paradigm shift in nearly all aspects of science. The first 15 years of data submissions were almost entirely gene microarrays, but submissions have slowly shifted to “next gen” sequencing data types. Just as RNA-seq has largely replaced gene microarray expression data, single-cell RNA-seq data are moving rapidly to capture a large portion of produced data.

Over this time, other data repositories have also grown in prominence; the cancer research data commons (CRDC) hosts a number of “data nodes”, each of which is largely devoted to a data modality [35,36], including imaging data (IDC) [37], proteomics data (PDC) [38], and the genomic data commons (GDC) [39], which is the source of the raw TCGA array data used in this study. The CRDC stores its data in the cloud, and there are a number of ways to access it, including using the ISB-CGC’s BigQuery data repository, where we acquired TCGA RNA-seq and CPTAC3 data [32,33].

In this project, we demonstrated the process of learning robust feature sets from “archived data” that can be used to train models and make predictions on contemporary data. Our models were able to reproduce cluster labels on known validation sources as well as new data, such as that from Tempus Labs. While we saw that the initial data-driven feature selection process did produce a feature set capable of identifying the protective clusters across modalities, it was improved through an interactive process of “feature refinement”, which included a “human-in-the-loop”.

However, working “across platforms” remains difficult. Gene microarrays and RNA-seq, while both providing quantitative measures of expression, have quite different measure characteristics. RNA-seq offers a wider dynamic range and greater sensitivity for detecting low-abundance transcripts, whereas microarray expression is constrained by background noise at the lower end and signal saturation at the upper end. Wolf et al. showed that gene expression variance was associated with gene length, nucleotide diversity, and the presence of non-coding RNA [40]. Depending on the features selected and their DNA coding properties, the predictive strength of certain feature pairs could be severely affected. Additionally, recent work demonstrates that the choice of normalization has an impactful effect on merging platforms successfully [41]. However, it needs to be remembered that in paired feature models, normalizing features across samples destroys the paired relationship, eliminating the information gain. Potentially, with the advent of deep learning methods, it may be possible to build an encoder–decoder system that could transfer data from either platform into the other platform or a shared latent space.

With data from multiple cohorts, it was clearly shown that deriving features from a single dataset can lead to overfitting; features that are highly informative within the training data are not at all informative in other patient cohorts. With the exception of fc3, which contained an especially strong signal, we found features in every other cluster that were predictive in TCGA but had no predictive power in the validation array data.

In Yang et al.’s [10] study, the number of samples is first divided by sex and then into five clusters. Each cluster therefore contains an extremely small number of patient samples. This fact makes training and working with predictive models difficult; the few examples within each cluster may lead to missing subtle signals, overfitting, and other predictive model problems. To some degree, these problems can be remedied by using robust ensemble statistical methods and a careful avoidance of overfitting, but there is often nothing better than additional data. Having matched data between array and RNA-seq platforms did help in performing feature refinement. However, even without matched samples, it may still be possible to expand the number of samples within each cluster by using methods such as cross-correlation analysis and deep learning techniques, perhaps to look at clinical and molecular associations simultaneously across datasets, expanding the ranks within each cluster and improving model performance. Additionally, with respect to contemporary diagnosis, cohorts should be created in order to homogenize on IDH1 status.

When model performance is considered, there was a sex-based difference; the female model appeared more robust and was validated using RNA-seq datasets. This difference in performance likely has many contributing factors. Initially, feature pairs were selected using a scoring heuristic, which described the degree to which the two features were separated in magnitude and by clustering purity. The female feature pairs tended to score much higher than feature pairs from males, expressing the difference in signal strength. The Yang et al. [10] clusters were each statistically associated with genes, forming a signature. The number of genes greatly varied across signatures; cluster mc4 was associated with only seven genes, while cluster mc5 was associated with 197 genes. In the female clusters, fc2 was associated with 21 genes, while fc3 was associated with 123 genes. This speaks to the relative difficulty of predicting cluster labels. The results shown in Yang et al.’s [10] work in their Figure 4A [10] show the disease-free interval for fc3. When compared to mc5, the protective cluster fc3 is clearly more separated from the other clusters.

Generally, it has been shown that IDH1 mutations have been associated with better outcomes. In the Yang et al. [10] cohort, IDH1 cases were more distributed across male cases and fc3 was associated with longer survival times regardless of IDH1 status, while IDH1 mutation stratified survival differences among mc5 cases. Similarly to what was previously found, where 70% of fc3 cases were of the IDH1 mutant, the predictive signature appeared to pick out primarily IDH1-mutant tumors in women. We saw that the REMBRANDT diagnosis was more weighted in oligodendroglioma and astrocytomas, both diseases that are known to harbor IDH1 mutations. On the other hand, the predictive signature in men, cluster label mc5, was not associated with IDH1 mutations, which was supported by the REMBRANDT diagnosis distribution being strongly weighted towards GBM, a disease identified by its association with the wildtype IDH1 status. Our signature clearly shows the sex-specific nature of the downstream molecular effects associated with the IDH1 mutation through clearly visible patterns of expression, which are not present in male patients.

Many of the genes selected in our curated set (Section 3.5) have been reported previously. If we first look at gene pair RBP1-BMP2, it has been found that RBP1 shows reduced expression in many prevalent cancers [42] and that in the Chinese Glioma Population Database (CGGA), RBP1 was one of the eight identified hub genes associated with IDH1 mutant status [43]. Also, when looking at associations between genetic aberrations and expression in gliomas, the methylation state of RBP1 was reported to be associated with the expression of BHLHE40 [44]. Also, BMP2 has been widely reported on, such as in correlation to poor prognosis in glioma patients [45] and part of a proposed glioma grading model [46].

In the gene pair PDPN-DLL3, we find two more well-known genes. PDPN has been reported to identify “a subset of aggressive and radiation-resistant glioblastoma cells” [47] and predict “poor prognosis in patients with glioma” [48] and that it “contributes to constructing immunosuppressive microenvironment in IDH wildtype glioma” [49]. DLL3 has also been reported on; it was found that high levels of the DLL3 protein is prognostic in IDH1 mutant gliomas but not in GBM, which tracks well in our results [50].

It has been reported that EMP3 has a multifunctional role in the regulation of membrane receptors associated with IDH wild-type glioblastoma [51], and that it potentially “mediates glioblastoma-associated macrophage infiltration to drive T cell exclusion” [52]. Both PDPN and EMP3 were found to be correlated with clinical outcomes in spheroid cultures derived from 20 glioblastomas [53].

Other fc3 genes in the curated set, FERMT1 [54], TMEM100 [55], DYNLT3 [56], EPHB1 [57], IGFBP2 [58], NNMT [59], and SH3GL2 [60] all show direct evidence to a relationship with glioma biology and patient prognosis.

While biomarkers to date have not been proven to be informative for treatment decisions, approaches such as these may provide for the identification of new markers and targets that may provide a therapeutic advantage. These informative genes have been previously identified in other studies and are found to be biologically important and predictive of patient prognosis. Still, several questions remain regarding the etiology of the sex specificity, the systems connection between them, and the pathways they reside in, and these warrant additional future research.

## 5. Conclusions

By using what might be considered “legacy data” and robust statistical prediction methods, we found that the female protective signature was able to be replicated in recently generated data from Tempus. However, we found that the predicted fc3 patients were largely composed of IDH1 mutants, confirming that the signature is associated with female IDH1 mutants. The same signature shows lesser predictive power in male patients, exemplifying the great importance of considering patient sex in disease studies, as other research has shown.

## 6. Methods

### 6.1. Data Sources

All data sources can be found in Supplemental Table S3. Raw array data for TCGA-GBM was downloaded from the GDC archive site as a collection of CEL files [39]. Additionally, raw CEL data were downloaded from NCBI GEO with accession numbers GSE13041, GSE16011, and REMBRANDT (GSE108474) and were processed as before. RNA-seq data were acquired through the ISB-CGC BigQuery tables [32].

### 6.2. Gene Microarray Data Processing

This work is based on a within-sample comparison of gene values. Most sources of Affymetrix array data use RMA normalization, which normalizes the gene expression measures across a set of samples, rendering the gene pair comparison invalid. This is due to the fact that instead of the feature logical *I*(*gene1* > *gene2*), it is instead *I*((*gene1*/*s1*) > (*gene2*/*s2*)) where *s1* and *s2* are scaling factors derived from the specific set of samples together. Thus, the relation has been altered by the data normalization.

Gene symbols were mapped to standard HGNC symbols using org.Hs.eg.db and internet resources such as GeneCards to differentiate when gene aliases pointed to more than one symbol [61]. In a number of cases, the array annotation did not have a clear gene symbol match and were labeled as unknown. Also, some genes have multiple symbols assigned, which are seen together; R replaces the “///” between symbols with “.....”, but these are indexed into the training data for cross-validation. Only the array data columns which were selected were scrutinized for the correct symbol.

The bioconductor R package SCAN.UPC was used to process the raw array data into sample specific normalized counts, meaning the normalization took place entirely within the sample [62]. No other processing or normalization was performed.

In the TCGA training array data, there are some samples that have multiple aliquots. Aliquots were selected based on the metadata contained in GBM.Gene_Expression.Level_1/data.freeze.txt, where some aliquots are marked as “non-canonical” and part of portioning studies. The lists of barcodes used are collected in the code repo and the patient samples were then matched to the cluster labels given by Yang et al. [10].

### 6.3. RNA-Seq Processing and Acquisition

De-identified RNA sequencing data (sequenced by Tempus) and de-identified clinical data from GBM patients were obtained from various academic medical centers around the United States (University of Michigan, University of Texas Southwestern, and Baylor College of Medicine). Tempus utilized exome capture RNA sequencing methodology using IDT xGen Lockdown Exome Probes [63]. Raw sequence reads were processed using the GDC mRNA Analysis Pipeline (https://github.com/NCI-GDC/gdc-rnaseq-cwl, accessed on 9 August 2022) [64].

### 6.4. Feature Selection

With processed array data, feature selection was performed using the TCGA-GBM training data. For each cluster (c1–c5) and patient sex (male or female), various types of information were calculated for each gene pair over all genes, including within-cluster expression averages, the distance between average levels, and most importantly, the proportion of samples with *gene1* > *gene2* for samples within the cluster and those outside the cluster. The difference between these proportions gives a metric called “Proportion Difference”. In order to limit results to predictive features, gene pairs that had a proportion difference less than 0.5 were filtered out, greatly reducing the potential feature space. For each gene pair, the ranking score was calculated using (Equation (1)) per cluster.

Feature ranking: Gene pairs were ranked by the score (Equation (1)), and features were selected by incrementally stepping down the ranking. A gene pair was selected when each member of the pair did not show a correlation with previously selected genes above 0.85. This eliminated gene pairs where one of the members had previously been selected. This produced the initial gene pair feature set.

Feature selection update: The initial set of features were updated after considering both the validation array data and the TCGA-GBM RNA-seq data. To incorporate information from the validation array data, the predicted cluster labels were used to compute proportion differences between the clusters, in the same manner as previously described with TCGA-GBM data. Then, gene pairs were rescored (Equation (2)) and the same selection procedure was followed. The TCGA RNA-seq was used to examine the correlation structure; gene pairs were filtered if they showed poor correlation.

### 6.5. Param Search

A simple grid search was performed over five parameters of the robencla model that control the classifier and the underlying XGBoost trees. They are as follows: max_depth (of the boosted trees), eta (learning rate), nrounds (number of rounds of training), lambda (L2 regularization in xgboost), and alpha (L1 regularization in xgboost). The optimal parameter set was selected through the maximization of the outcome of interest, which was the average F1 classification metric over clusters using TCGA-GBM cross-validation.

Overall, the parameter search resulted in the following parameter values: The ensemble size was set to 11 (for example, there are 11 XGBoost binary classifiers for predicting each cluster) and the tree depth was set to 12. Each member of the ensemble is trained on a randomly sampled 80% portion of data, and the median cluster label prediction is taken from within the ensemble.

### 6.6. Survival Curves

The R packages “survival” and “survminer” were used to create survival models and produce survival curve plots. The model was specified as “survfit(Surv(Survival, CensoredCode)~BestCalls, data=df)”.

### 6.7. Classification Task Order

CV on TCGA-GBM in the model is trained using TCGA-GBM data in a 10-fold cross-validation. Predictions from each fold are collated, and classification metrics were computed from that results table.

Classification on the additional validation array datasets used the initial gene pairs feature set and TCGA-GBM array data to make cluster label predictions on the validation array data. The clinical data from the three sources were harmonized to overall survival in days, and survival plots were made.

Model trained on validation array data: validation array data from all three sources were used with the initial gene pairs feature set to make predictions back to the known TCGA-GBM cluster labels.

TCGA-GBM RNA-seq: The TCGA-GBM was used for training, and the model was used to predict the known cluster labels across expression platforms.

TEMPUS RNA-seq: The TCGA-GBM array data were used for training with the updated feature set and the model was used to predict cluster labels on samples from TEMPUS-GBM. TEMPUS clinical data were used to plot survival curves.

CPTAC3 RNA-seq: The TCGA-GBM was used for training, and the model was used to predict cluster labels on samples from CPTAC-3-GBM. Clinical data were used to plot survival curves.

### 6.8. Feature Alignment

For each of the four female datasets (TCGA-GBM array, TCGA-GBM RNA-seq, Validation array data, TEMPUS), a model was trained using either the known cluster labels or cluster labels as predicted by a model trained on TCGA-GBM array data. Then, the top 10 most important features were extracted from each of the four datasets. The features were examined and selected if they were found in at least three of the datasets (always including the TCGA-GBM array data).

### 6.9. Single-Cell RNA-Seq

Data were downloaded from the UCSC CellBrowser and loaded into an AnnData object using Scanpy [65]. This was a total of 223,113 cells and 29,661 genes. Both dotplots and stacked violin plots were produced with Scanpy.

### 6.10. Code Repository

All data, scripts, and figures can be found at https://github.com/Gibbsdavidl/GBM_Sex_Specific_Cluster_Prediction (accessed on 20 January 2024).

## Figures and Tables

**Table 1:** Aggregated gene pair feature set.

ClusterLabel	GeneA	GeneB	Datasets	Array PD (A < B)	RNA-Seq PD (A < B)
cluster1	POSTN	C1QL1	3	−0.51	−0.46
cluster1	CD46	PDGFRA	3	−0.6	−0.36
cluster1	ACSL3	TF	3	−0.51	−0.42
cluster1	DCX	LGR4	2	0.61	0.38
cluster1	EGFR	PMP2	2	−0.52	−0.27
cluster1	TMSB15A	LXN	2	0.53	0.48
cluster1	TCEAL2	MYO5C	2	0.5	0.44
cluster2	HILPDA	BANF1	3	−0.67	−0.59
cluster2	NDRG1	APLP2	3	−0.7	−0.61
cluster2	IFITM3	BNIP3	2	0.57	0.53
cluster2	MRFAP1L1	ZNF395	2	0.68	0.48
cluster3	PDPN	DLL3	3	0.9	0.56
cluster3	TMEM100	DYNLT3	3	−0.86	−0.81
cluster3	EMP3	FERMT1	3	0.7	0.53
cluster3	RBP1	BMP2	2	0.88	0.64
cluster3	EPHB1	IGFBP2	2	−0.75	−0.64
cluster3	NNMT	SH3GL2	2	0.71	0.5
cluster4	P4HA1	LMO3	3	0.61	0.57
cluster4	CDKN2A	MYC	3	−0.51	−0.3
cluster4	MAOB	VEGFA	3	−0.61	−0.6
cluster4	C21orf62	SEMA5A	2	−0.79	−0.77
cluster4	APLNR	PHLDA1	2	−0.64	−0.7
cluster5	GULP1	CA10	3	0.69	0.69
cluster5	SLC7A11	NKX2-2	3	0.57	0.41
cluster5	MYO5C	UGT8	3	0.58	0.48
cluster5	C1QL1	LTBP1	3	−0.56	−0.49
cluster5	ECM2	GPR17	2	0.51	0.32

Three datasets include TCGA, validation arrays, TCGA RNA-seq, and *PD* (Equation (1)) values for TCGA data.

## Data Availability

Some restrictions apply to the availability of these data. The datasets used to conduct this study were provided through an agreement with Tempus. Requests for access can be made at https://www.tempus.com/contact-us (accessed on 9 August 2022). Publicly available data and code can be found at https://github.com/Gibbsdavidl/GBM_Sex_Specific_Cluster_Prediction (accessed on 20 January 2024).

## Supplementary Materials

The following supporting information can be downloaded at: https://www.mdpi.com/article/10.3390/cancers17030445/s1, Figure S1: Yang et al. [10] signatures overlap; Figure S2: TCGA RNA-seq results before and after the feature alignment; Figure S3: Refined features made suitable predictions on TEMPUS data; Table S1: Software parameters list; Table S2: Cross validation results on TCGA; Table S3: List of data sources.
