# Supplementary figures and images for: Robust Cluster Prediction Across Data Types Validates Association of Sex and Therapy Response in GBM
Source: Cancers (Basel). 2025 Jan 28;17(3):445. doi: 10.3390/cancers17030445 (PMC11815886; doi:10.3390/cancers17030445)

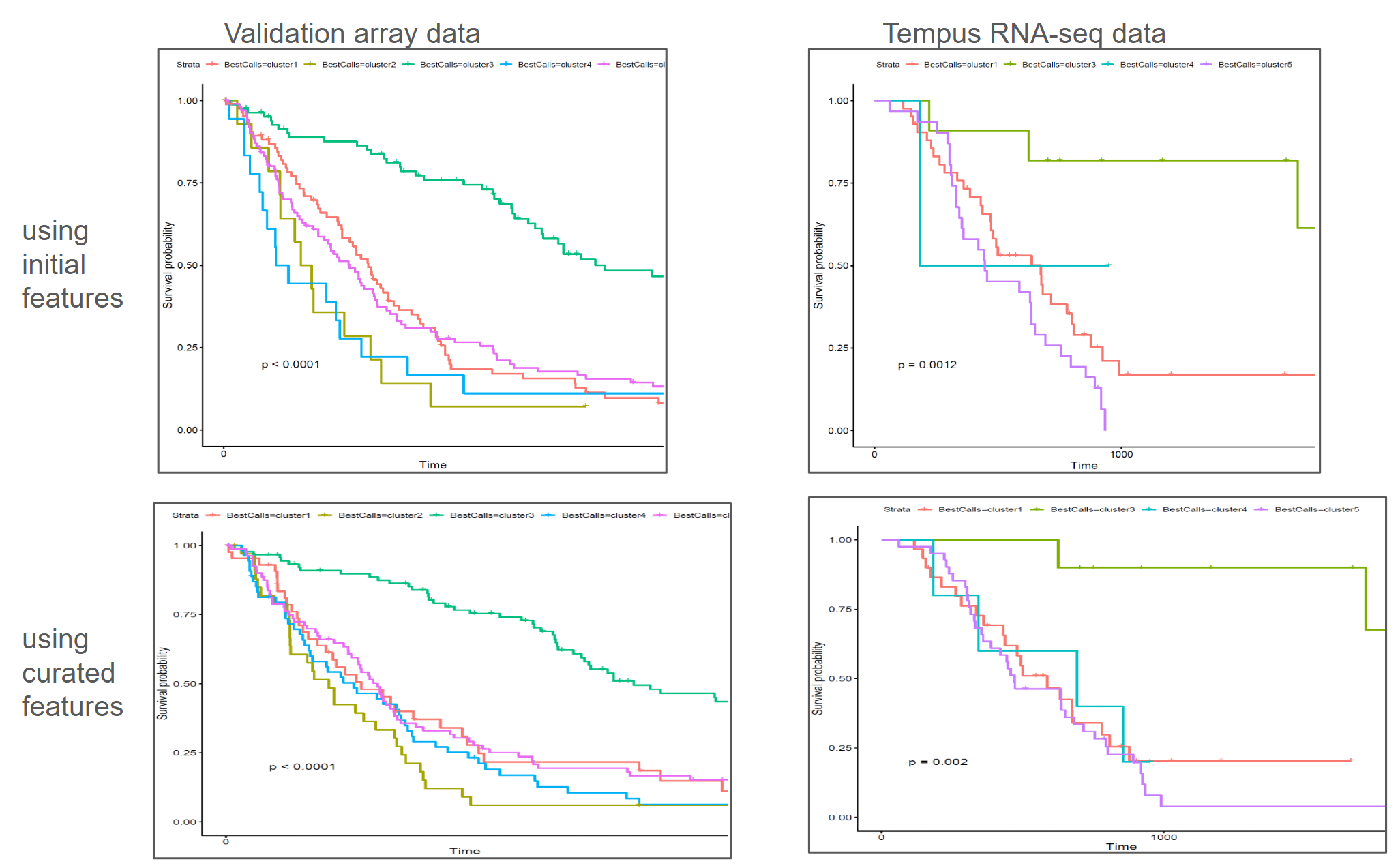

Supplement: Supplementary file 1 [file cancers-17-00445-s001.zip › Supplemental Figure S1.png]

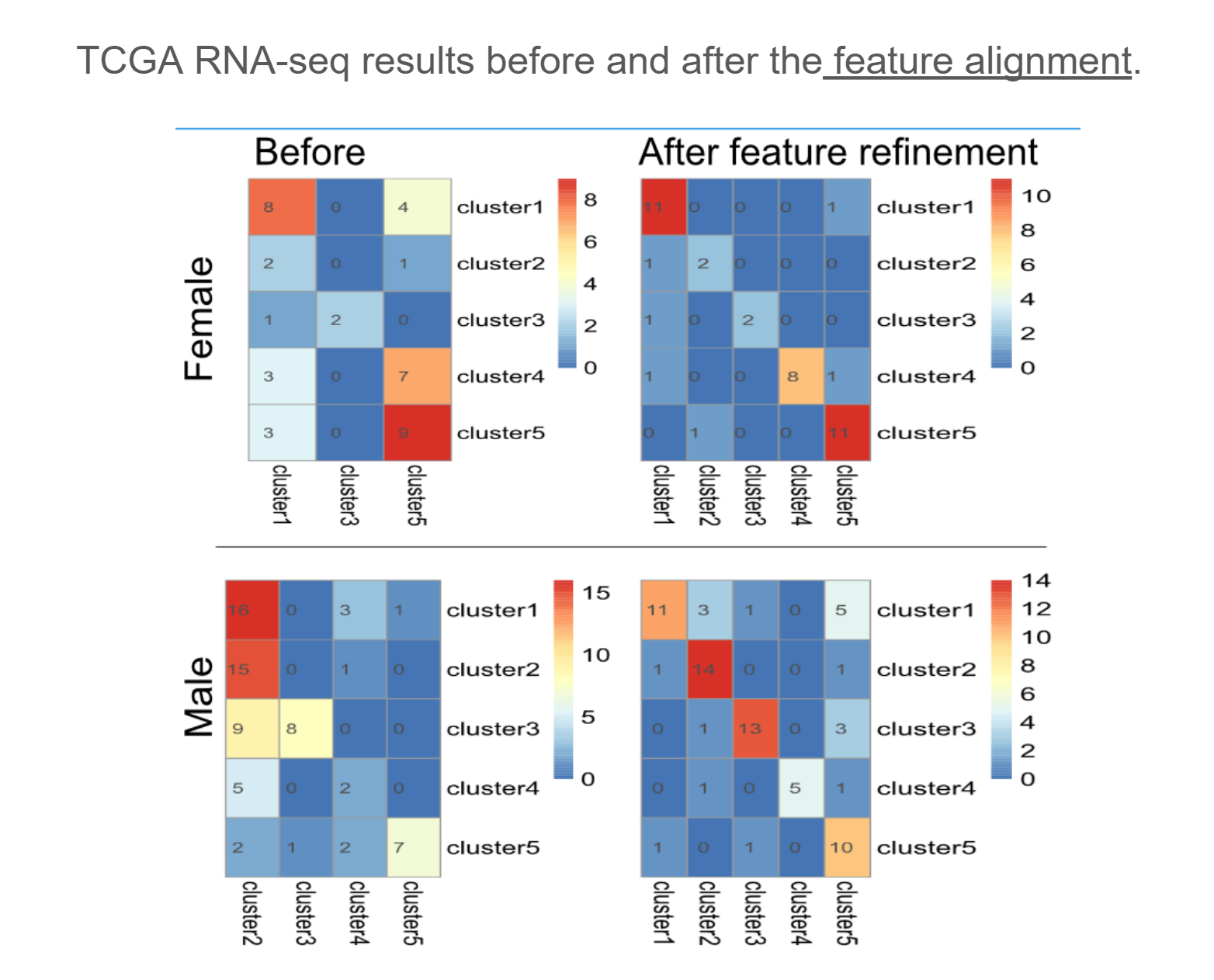

Supplement: Supplementary file 1 [file cancers-17-00445-s001.zip › Supplemental Figure S2.png]

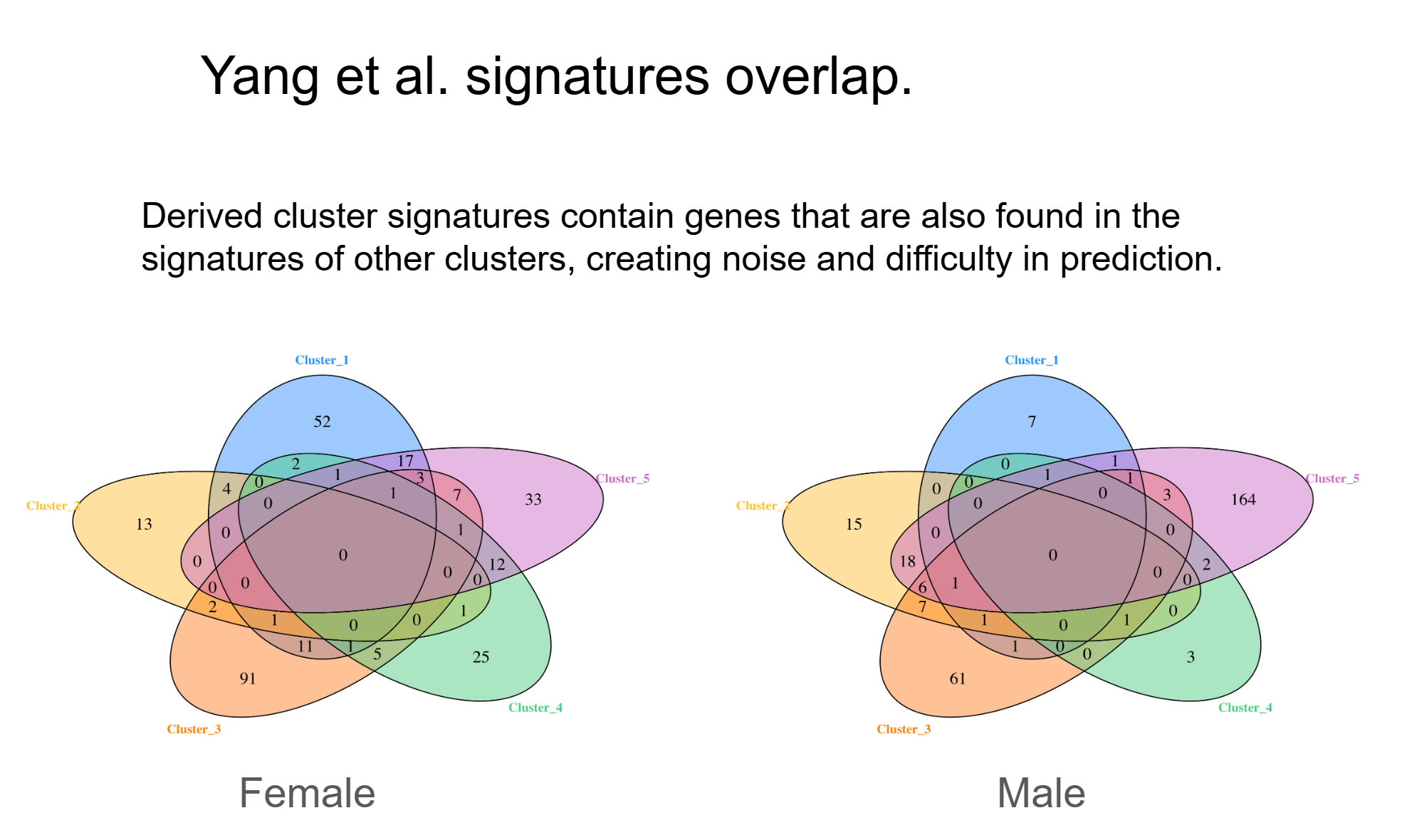

Supplement: Supplementary file 1 [file cancers-17-00445-s001.zip › Supplemental Figure S3.png]
